# Draft Genome Sequence of an Acid-Tolerant Yeast, *Candida zemplinina* NP2, a Potential Producer of Organic Acids

**DOI:** 10.1128/genomeA.01052-17

**Published:** 2017-09-28

**Authors:** Hyeok-Jin Ko, Hyun Joo Park, Sun Hee Lee, Haeyoung Jeong, Jung-Hoon Bae, Bong Hyun Sung, In-Geol Choi, Jung-Hoon Sohn

**Affiliations:** a Cell Factory Research Center, Korea Research Institute of Bioscience and Biotechnology (KRIBB), Daejeon, Republic of Korea; b Infectious Disease Research Center, Korea Research Institute of Bioscience and Biotechnology (KRIBB), Daejeon, Republic of Korea; c Department of Biotechnology, College of Life Sciences and Biotechnology, Korea University, Seoul, Republic of Korea

## Abstract

Here, we report the draft genome sequence of the acid-tolerant yeast *Candida zemplinina* NP2, which was isolated from peach peels. This genome sequence will aid in the understanding of the organism’s physiological properties as a potential producer of organic acids in acidic environments.

## GENOME ANNOUNCEMENT

*Candida zemplinina* is synonymously known as *Starmerella bacillaris* and is an acidophilic yeast species frequently isolated from fruit sources and wine environments (1). Owing to its osmotolerant, psychrotolerant, and acid-tolerant properties (2, 3), *C. zemplinina* has been applied in mixed fermentation with *Saccharomyces cerevisiae* to reduce acetic acid byproducts generated by *S. cerevisiae* in botrytized wine fermentation (4). Recently, organic acids, such as succinic acid, 3-hydroxypropionic acid, and lactic acid, have become increasingly attractive as important platform chemicals in biorefining applications (5). With an ability to grow at a low pH (pH < 2), *C. zemplinina* is explored for its potential as the producer of a variety of organic acids. Here, we report the draft genome sequence of *C. zemplinina* NP2, an acid-tolerant yeast isolated from peach peels, in order to obtain further insight into the acid-tolerant properties of NP2 in fermentation of a variety of organic acids under acidic conditions.

Genome sequencing of *C. zemplinina* NP2 was performed by the Illumina HiSeq 2500 platform using paired-end libraries at the Core Facility Management Center in the Korea Research Institute of Bioscience and Biotechnology (KRIBB). We obtained 15.6 million paired-end reads, with 78.9-fold coverage. Low-quality (*Q* < 30) reads were identified and eliminated by the HTQC program (6). *De novo* assembly of the filtered reads was performed using Velvet, version 1.2.10, and VelvetOptimiser, version 2.2.5 (7). The final assembly was 9,311,634 bp, with 117 scaffolds and 145 gaps. The G+C content was 39.46%, and 131 tRNA-coding sequences, including 2 tRNA-like pseudogenes, were identified by tRNAscan-SE (8). The *N*_50_ value was 481,187 bp, and the length of the longest contig was 1,363,694 bp. Gene prediction of the genome sequence was performed using AUGUSTUS with a training set of *Candida albicans* (9). In total, 3,741 protein-coding genes were predicted in the draft genome, and 3,285 of these candidates were functionally characterized using an InterProScan search against the InterPro protein signature databases (10).

### Accession number(s).

The nucleotide sequence has been deposited in GenBank under the accession number NQLE00000000.
